# Dietary oxalate-calcium balance and the incidence of hypertension and chronic kidney disease: a prospective study among an Asian population

**DOI:** 10.1186/s12986-022-00709-w

**Published:** 2022-11-03

**Authors:** Parvin Mirmiran, Zahra Bahadoran, Fereidoun Azizi

**Affiliations:** 1 Nutrition and Endocrine Research Center, Research Institute for Endocrine Sciences, Shahid Beheshti University of Medical Sciences, No. 24, Shahid-Erabi St., Yeman St., P.O. Box 19395-4763, Velenjak, Tehran, Iran; 2 Endocrine Research Center, Research Institute for Endocrine Sciences, Shahid Beheshti University of Medical Sciences, Tehran, Iran

**Keywords:** Oxalate, Calcium, Hypertension, Estimated glomerular filtration rate, Chronic kidney disease

## Abstract

**Background:**

The potential effects of dietary oxalate (Ox) intake on cardio-renal function have remained unestablished. We evaluated the effect of usual Ox intake and its interaction with dietary calcium (Ca) on incident hypertension (HTN) and chronic kidney disease (CKD).

**Methods:**

Adult men and women, free of HTN and CKD at baseline (2006–2008), were recruited. Dietary intakes were assessed using a validated food frequency questionnaire, and the outcomes were documented up to 2014–2017. Multivariate Cox proportional hazard regression models were used to estimate the development of HTN and CKD in relation to Ox intakes. Repeated-measures generalized estimating equation (GEE) linear regression models were used to assess possible effect of Ox-intake on the estimated glomerular filtration rate (eGFR) and blood pressure levels over eight years.

**Results:**

Dietary Ox intakes were positively associated with incident CKD (HR = 2.59, 95% CI = 1.46–4.64) and HTN (HR = 1.79, 95% CI = 1.05–3.04). Compared to high-Ca consumers, subjects who had lower Ca intakes (< 990 *vs.* 1580 mg/d) had a higher incidence of CKD and HTN (HR = 2.43, 95% CI = 1.06–5.55, and HR = 1.72, 95% CI = 0.76–3.78). Participants with higher intakes of Ox (> 220 *vs.* < 150 mg/d) had lower eGFR values (75.3, 95% CI = 75.0–76.5 *vs.* 77.3, 95% CI = 76.6–78.1 mL/min/1.73m^2^, *P*_time×group_ = 0.004) and higher SBP levels (112, 95% CI = 111–113 *vs.* 109, 95% CI = 108–110 mmHg, *P*_time×group_ = 0.007) overtime.

**Conclusion:**

Higher dietary Ox intake may increase the risk of HTN and CKD. The relation between dietary Ox and risk of HTN and CKD seems to be varied by Ca intake, and subjects with lower Ca intakes may be more burdened by excessive amounts of dietary Ox.

## Introduction

Oxalate (Ox) is a potentially toxic metabolite eliminated primarily by glomerular filtration and tubular secretion [1]. In mammals, ascorbic acid and glyoxylate account for half the total endogenous Ox production, and the remaining is derived from glycolate- and glyoxylate-forming reactions and dietary and other endogenous sources [1]. An excessive amount of dietary Ox has been considered the leading cause of secondary hyperoxaluria [2, 3] and a risk factor for developing systemic oxalosis and vascular and renal dysfunction [4]. Moreover, a high-Ox diet may adversely affect vascular function by impaired oxidant-antioxidant balance, induction of inflammation, and endothelial cell toxicity [5–10]. A higher load of Ox intake has also been linked to calcium (Ca)-Ox nephrolithiasis, acute and chronic kidney disease (CKD) [11], and elevated Ox excretion (≥ 27.8 vs. < 11.5 mg/d), increasing the risk of CKD progression by 33% [12].

The amount of Ox in a regular diet has been reported in a range of 50–350 mg/d [13, 14]; however, it may surpass 1000–2000 mg/d when Ox-rich foods (i.e., spinach, rice bran, tea, nuts, chocolate, and rhubarb [15]) are highly consumed [16, 17]. A non-linear association was observed between dietary and urinary Ox, and each 100 mg of Ox intake corresponds to increased urinary Ox excretion by ~ 3 mg (within a range of 50–750 mg/day) in a diet containing 1000 mg Ca [18].

No evidence is available regarding the possible association between dietary Ca intake and the risk of developing CKD, however a usual intake of 800–1000 mg/day of Ca is recommended to achieve a neutral Ca balance and avoid adverse effects of either negative or positive  Ca balance in CKD [19]. Meta-analysis of cohort studies reported that dietary Ca intake was slightly associated with a reduced risk of HTN (a 7% decrease in the risk of HTN per every 500 mg/d increments of dietary Ca) [20], probably via decreasing Ox absorption.

An imbalanced diet in Ca-Ox accelerates gastrointestinal (GI) absorption of dietary Ox and induces hyperoxaluria [21, 22]. Since the amount of GI-absorbed Ox is modulated by the dietary Ca [21–23], the dietary Ca-to-Ox ratio would be theoretically a better predictor of renal and vascular dysfunction rather than Ox per se, a hypothesis that is remained uninvestigated.

The possible effect of the dietary load of Ox and the balance of dietary Ca-Ox on cardio-renal function and blood pressure homeostasis has not yet been investigated in a population-based setting. Here, we evaluated longitudinal associations of dietary Ox and its potential interaction with Ca intake levels with the incident HTN and CKD in a free-living Asian population with a high prevalence and incidence of cardiovascular diseases.

## Methods

### Study population

**Fig. 1:**
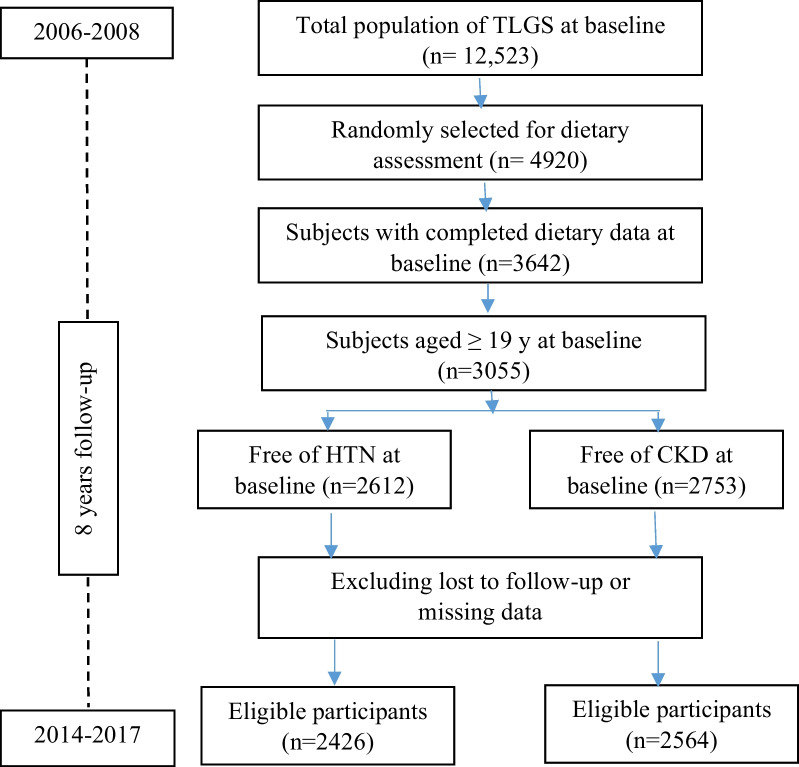
Study flowchart

Study participants of the current study were recruited from the Tehran Lipid and Glucose Study (TLGS). Details of the TLGS rationale and design, and study population have been reported extensively elsewhere [24]. In brief, the TLGS started in 1999 in a large-scale community-based prospective study on 15,005 individuals aged ≥ 3 years, a representative sample of residents of district 13 of Tehran, the capital city of Iran [25]. The measurements are repeated at 3-year intervals to assess changes in non-communicable diseases (NCDs) risk factors. The current analyses were conducted on available data of adult men and women (≥ 19 y) who participated in the third TLGS examination (2006–2008), with completed demographics, anthropometric, and biochemical measurements (subjects with missing data were excluded from the study). Two separate lines of exclusions were carried out for the outcomes; prevalent cases of CKD and HTN were excluded at baseline, and the remaining eligible participants were followed up to the sixth TLGS examination (2015–2017). The study flowchart of the participants is provided in Fig. 1.

We obtained written informed consent from all participants. Based on the ethical guidelines of the 1975 Declaration of Helsinki, the study protocol was approved by the Ethics Research Council and the Scientific Committee of the Research Institute for Endocrine Sciences, Shahid Beheshti University of Medical Sciences. The ethics code and registration ID of the study protocol are IR.SBMU.ENDOCRINE.REC.1400.039 and 29,160, respectively.

### Demographic, anthropometric, and biochemical measurements

Details of data collection and measurements of the variables in the TLGS have been reported elsewhere [26]. Systolic (SBP) and diastolic (DBP) blood pressures were measured using a standard mercury sphygmomanometer calibrated by the Institute of Standards and Industrial Research of Iran [27]. Blood pressure was measured twice on the participants' right arm after a 15-min rest in a sitting position, with at least a 30-s interval between the two measurements. The mean of the two measurements was considered as the participant’s blood pressure. Details of biochemical measurements in the TLGS samples have been described in detail elsewhere [28]. In brief, measurements of fasting serum glucose (FSG), triglyceride (TG), and high-density lipoprotein cholesterol (HDL-C) levels were all done after a 12-to 14-h overnight fast. The standard oral glucose tolerance test (OGTT) was performed for all participants who were not on glucose-lowering medications.

### Dietary assessment

Information on dietary intake at baseline (2006–2008) was collected using a validated 168-item food frequency questionnaire (FFQ). Details of dietary assessment have been reported elsewhere [29]. In brief, trained dietitians with at least five years of experience in the TLGS survey asked participants to designate their intake frequency for each food item consumed during the past year on a daily, weekly, or monthly basis. Portion sizes of consumed foods reported in household measures were then converted to grams [30]. Reliability, relative validity [31], and stability of data retrieved by the FFQ over time [32] were evaluated and reported elsewhere. In brief, the validity of the FFQ was confirmed by comparing food groups and nutrient values determined from the questionnaire with values estimated from the average of twelve 24-h dietary recall surveys, and the reliability has been assessed by comparing energy and nutrient intakes from two FFQs; Pearson correlation coefficients and intra-class correlation for energy and nutrients showed acceptable agreements between FFQ and twelve 24-h dietary recall surveys, and FFQ1 and FFQ2 [31].

Since the Iranian Food Composition Table is incomplete and has limited data on raw foods and beverages' nutrient content, the US Department of Agriculture Food Composition Table was used to determine energy and nutrients intakes of the participants [19]. The Ox contents of foods were derived from available databases of Ox [16, 17, 33]. Estimated dietary Ox intakes were computed from the reported frequency of consumption of each specified unit of foods containing Ox. A residual adjustment was performed using a regression model, with total caloric intake as the independent variable and total Ox intake as the dependent variable [34].

### Definition of outcomes and terms

Details of the outcomes measurement and confirmation in the TLGS have been described in detail elsewhere [35]. In brief, all outcomes were confirmed by an expert medical physician and the outcome committee. The HTN was defined as SBP ≥ 140 or mm Hg DBP ≥ 90 mmHg or self-reported taking blood pressure-lowering medications [36]. The CKD Epidemiology Collaboration (EPI) equation was used to calculate the estimated glomerular filtration rate (eGFR). As a single equation CKD-EPI has been expressed as follows: eGFR = 141 × min (S_cr_/κ, 1)^α^ × max (S_cr_/κ, 1)^−1.209^ × 0.993^age^ × 1.018 [if female] × 1.159 [if black]. In this equation, S_cr_ is serum creatinine in mg/dL; κ is 0.7 and 0.9 for men and women, respectively, α is − 0.329 and − 0.411 for men and women; min indicates the minimum of S_cr_/κ or 1, and max indicates a maximum of S_cr_/κ or 1 [37]. Incident CKD was defined as eGFR < 60 mL/min/1.73 m^2^ occurring at any time during the follow-up period; this corresponds to stage 3 to stage 5 CKD based on the Kidney Disease Outcomes and Quality Initiative guidelines [38].

Type 2 diabetes mellitus (T2DM) was defined as fasting serum glucose ≥ 126 mg/dL, 2-h serum glucose ≥ 200 mg/dL, or use of anti-diabetic medications [39].

### Statistical analyses

All statistical analyses were performed using the Statistical Package for Social Science (version 20; IBM Corp., Armonk, NY, USA) and MedCalc Statistical Software version 15.8 (MedCalc Software bvba, Ostend, Belgium). A *P*-value < 0.05 is considered significant.

Mean and standard deviation (SD) of values and the frequency (%) of the baseline participants’ characteristics were compared between subjects with and without incidence of HTN and CKD using an independent sample t-test or Chi-square test. The Mood’s median test was used to compare the median Ox intake between groups.

Cox proportional hazards regression models with person-year as the underlying time metric were used to estimate hazard ratios (HRs) and 95% confidence intervals (CIs) for the association between dietary Ox intakes (as a log-transformed variable) and the outcomes.

We also tested the hypothesis that the relationship between dietary Ox and developing HTN and CKD varied by Ca intake. HRs (95% CI) of HTN and CKD were estimated across different levels of Ca intake (i.e., 1180 < and > 1180 mg/d as median intake of the population). The survival time was the interval between the first and the last observation dates for the censored and lost to follow-up subjects. Follow-up duration and person-years were calculated using the measured survival time. The proportional hazard assumption of the multivariable Cox model was assessed using Schoenfeld’s global test of residuals. A list of potential confounding variables was derived from previous studies; a univariate analysis was performed for potential confounding variables, and those with *P*_E_ < 0.2 were selected for the final multivariable model; *P*_E_ (*P*-value for entry) determines which variables should be included in the multivariable model [40]. Potential confounding variables finally adjusted in the cox models included age (as two categories of < and ≥ 65 y) (according to a previous study of our research group among TLGS dataset) [41], sex (male/female), BMI (kg/m^2^), current smoking (yes/no), systolic blood pressure (mmHg), T2DM (yes/no), eGFR (mL/min/1.73m^2^, for HTN only), total daily calorie intake (Kcal/day), total dietary fats (g/d), and total fiber intakes (g/d). For CKD, model 1 was adjusted for age and sex; model 2 was additionally adjusted for systolic blood pressure, type 2 diabetes, serum creatinine, body mass index, and smoking; model 3 was additionally adjusted for total energy intakes, dietary intakes of total fats, fiber. For HTN, model 1 was adjusted for age and sex; model 2 was additionally adjusted for type 2 diabetes, eGFR, body mass index, and smoking; model 3 was additionally adjusted for total energy intakes, dietary intakes of total fats, and fiber.

The possible effect of Ox-intake levels (across tertile categories, i.e., < 150, 150–220, and > 220 mg/d) on eGFR, SBP, and DBP levels over eight years of follow-up, the estimated overall mean of the variables between groups, were compared using repeated-measures generalized estimating equation (GEE) linear regression models, with controlling correlation among observations repeated for four times (baseline, first, second and third examinations) were used.

The receiver operator characteristic (ROC) curve analysis was used with an estimation of the variable sensitivity and specificity to determine the cut-off point of dietary Ox-to-Ca ratio for the risk of developing HTN and CKD. The cut-off point was assessed by the maximum value of sensitivity + specificity – 1 (Youden index); the index is preferable for the finding of the optimal cut-off point because it is clinically translated to maximizing correct classification and minimizing misclassification rates [42].

## Results

Final analyses were performed on data of 2564 and 2426 participants for CKD and HTN, respectively. The median follow-up period was 8.4 y (IQR = 5.8–9.4 y) and 8.3 (IQR = 4.9–9.4 y) for incident CKD and HTN, respectively. The median (IQR) of dietary Ox intake and mean (SD) Ox-to-Ca ratio was 182 (133–244 mg/d) and 0.17 (0.07) in the whole population. The incidence rate of HTN and CKD was 24.4 and 20.7% upon an 8-year follow-up. Baseline characteristics and distributions of the major known cardiometabolic risk factors across outcome status of the participants (with and without incidence of HTN and CKD) are shown in Table 1. Incident- compared to non-incident cases of both CKD (0.18 ± 0.08 *vs.* 0.17 ± 0.09) and HTN (0.19 ± 0.09 *vs.* 0.17 ± 0.19) were more likely to have higher dietary Ox-to-Ca ratio. Table 2 shows baseline characteristics of the study participants across tertile categories of Ox intake.

**Table 1:** Baseline characteristics of the study participants

	Incident-CKD *(n* = *530)*	Non-CKD *(n* = *2034)*	Incident-HTN *(n* = *591)*	Non-HTN *(n* = *1835)*
Age *(y)*	50.6 ± 11.1	34.5 ± 11.2**	46.4 ± 12.7	34.9 ± 12.0**
Male *(%)*	47.6	42.8*	48.4	42.2**
Smoking *(%)*	8.9	10.1	8.3	10.3
BMI *(m*^*2*^*/kg)*	28.4 ± 4.5	26.5 ± 4.8**	29.0 ± 4.7	25.9 ± 4.6**
SBP *(mm Hg)*	72.8 ± 8.2	70.7 ± 9.1**	76.7 ± 7.7	69.5 ± 8.6**
DBP *(mm Hg)*	110 ± 12.6	107 ± 12.0**	117 ± 11.3	105 ± 11.2**
FSG *(mm Hg)*	98.5 ± 32.5	88.8 ± 17.4**	97.2 ± 30.5	87.3 ± 14.5**
Serum TG *(mg/dL)*	165 ± 93.8	135 ± 83.1**	167 ± 94.0	127 ± 77.1**
HDL-C *(mg/dL)*	42.2 ± 10.6	42.6 ± 10.1	41.1 ± 9.5	43.3 ± 10.4**
LDL-C *(mg/dL)*	123 ± 33.5	111 ± 31.9*	121 ± 36.5	111 ± 31.9*
Serum Cr *(mg/dL)*	1.06 ± 0.14	1.02 ± 0.14**	1.04 ± 0.14	1.03 ± 0.14
eGFR (*mL/min/1.73m*^*2*^*)*	69.5 ± 8.1	83.1 ± 11.4**	77.3 ± 10.5	82.2 ± 12.1*
T2DM *(%)*	14.0	4.2**	11.4	2.7**
Dietary Na–K ratio	1.29 ± 1.05	1.35 ± 1.21	1.31 ± 0.99	1.35 ± 1.21
Dietary Ox *(mg/d)*^*†*^	187 (144–257)	180 (130–243)	190 (133–257)	177 (130–241)**
Dietary Ca *(mg/d)*	1212 ± 459	1220 ± 462	1195 ± 457	1213 ± 467
Dietary Ox-to-Ca ratio	0.18 ± 0.08	0.17 ± 0.09**	0.19 ± 0.09	0.17 ± 0.19**

Data are mean ± SD unless stated otherwise (independent t-test and chi-square test were used for continuous and dichotomous variables, respectively

*CKD* chronic kidney disease; *HTN* hypertension; *BMI* body mass index; *SBP* systolic blood pressure; *DBP* diastolic blood pressure; *FSG* fasting serum glucose; *TG* triglycerides; *HDL-C* high-density lipoprotein cholesterol; *LDL-C* low density lipoprotein cholesterol; *Cr* creatinine; *eGFR* estimated glomerular filtration rate; *Ox* oxalate; *Ca* calcium; *Na* sodium; *K* potassium

^†^Median (inter-quartile range, IQR); the Mood’s median test was used to compare the median of Ox intakes between groups

*P < 0.05

**P < 0.01

**Table 2:** Baseline characteristics of the study participants across tertile categories of Ox intake

	Dataset of CKD			Dataset of HTN		
	Tertile 1	Tertile 2	Tertile 3	Tertile 1	Tertile 2	Tertile 3
Case/total *(n)*	146/854	199/855	185/855	190/808	182/809	219/809
Age *(y)*	35.1 ± 12.2	38.3 ± 13.2^a^	39.4 ± 13.0^a^	36.4 ± 12.8	37.4 ± 12.9	39.3 ± 13.4
Male *(%)*	41.1	47.5	51.2^*^	38.5	43.0	49.6*
Smoking *(%)*	12.7	11.6	14.9^*^	9.1	7.8	11.6
BMI *(m*^*2*^*/kg)*	26.5 ± 4.8	26.8 ± 4.7	27.3 ± 4.9^a^	26.3 ± 4.7	26.6 ± 4.7	27.0 ± 4.8^a^
SBP *(mm Hg)*	106 ± 12.1	108 ± 11.8^a^	108 ± 12.4^a^	106 ± 12.5	108 ± 11.8^a^	109 ± 12.9^a,b^
DBP *(mm Hg)*	70.5 ± 9.1	71.5 ± 8.6^a^	71.2 ± 9.1	70.8 ± 9.2	71.5 ± 8.7	71.5 ± 9.1
FPG *(mm Hg)*	88.9 ± 18.2	90.1 ± 18.5	93.7 ± 27.1^a,b^	88.4 ± 17.2	88.9 ± 17.2	91.8 ± 24.7^a,b^
Serum TG *(mg/dL)*	134 ± 86	142 ± 85	146 ± 86^a,b^	83.7 ± 2.9	84.8 ± 2.9	81.3 ± 2.8^a^
HDL-C *(mg/dL)*	43.0 ± 10.6	42.3 ± 10.1	42.4 ± 9.9	43.2 ± 10.7	42.8 ± 10.2	42.4 ± 9.8
LDL-C *(mg/dL)*	111 ± 30.7	113 ± 32.2	117 ± 34.6^a,b^	112 ± 31.4	113 ± 32.3	117 ± 35.3^a,b^
Serum Cr *(mg/dL)*	1.02 ± 0.14	1.03 ± 0.13^a^	1.04 ± 0.14^a^	1.01 ± 0.14	1.03 ± 0.14	1.04 ± 0.14^a^
eGFR (*mL/min/1.73m*^*2*^*)*	81.3 ± 11.9	80.2 ± 12.1^a^	79.6 ± 12.0^a^	81.7 ± 11.5	81.2 ± 12.1	80.3 ± 12.1^a^
T2DM *(%)*	5.6	5.5	7.7	5.1	4.3	5.3
Total Ox^†^ *(mg/d)*	117 (97.7–133)	182 (164–198)	278 (246–339)	115 (95–131)	180 (163–197)	278 (245–340)

*CKD* chronic kidney disease; *HTN* hypertension; *BMI* body mass index; *SBP* systolic blood pressure; *DBP* diastolic blood pressure; *FPG* fasting plasma glucose; *TG* triglycerides; *HDL-C* high-density lipoprotein cholesterol; *LDL-C* low density lipoprotein cholesterol; *Cr* creatinine; *eGFR* estimated glomerular filtration rate

Data are mean ± SD unless stated otherwise ^†^ Median (inter-quartile range, IQR)

^*a*^Different from first tertile (*P* < 0.05), analysis of variance (ANOVA) was used

^b^Different from second tertile (*P* < 0.05), analysis of variance (ANOVA) was used

*Significant difference across group (chi-square test were used)

Hazard ratios (95% CI) of HTN and CKD outcomes in relation to dietary Ox intakes are reported in Table 3. In the fully adjusted Cox proportional hazards model, we observed a significantly elevated risk of CKD (HR = 2.59, 95% CI = 1.46–4.64) and HTN (HR = 1.79, 95% CI = 1.05–3.04) head-to-head of increased dietary Ox. Compared to high-Ca consumers, subjects with lower Ca intake (< 1180 mg/d) had a higher risk of both CKD (HR = 2.43, 95% CI = 1.06–5.55 *vs.* HR = 1.72, 95% CI = 0.76–3.78) and HTN (HR = 2.68, 95% CI = 1.10–6.49 *vs.* HR = 1.48, 95% CI = 0.72–3.04). No association was observed between dietary Ox and the outcomes in subjects who consumed dietary Ca above the median. The *P* value for interaction between Ox and Ca intake was 0.002 and 0.019 for CKD and HTN, respectively.

**Table 3:** The hazard ratio (95% CI) of chronic kidney disease and hypertension in relation to dietary oxalate and oxalate-to-calcium ratio

	CKD	HTN
*Dietary Ox (mg/d)*		
*Crude*	1.89 (1.25–2.85)	1.68 (1.16–2.65)
*Model 1*	1.79 (1.18–2.71)	1.52 (1.04–2.22)
*Model 2*	1.55 (0.95–2.55)	1.18 (0.77–1.83)
*Model 3*	2.59 (1.46–4.64)	1.79 (1.05–3.04)
Dietary Ox (mg/d)^*^Ca (mg/d)^*†*^		
*Low-Ca diet*	2.43 (1.06–5.55)	2.68 (1.10–6.49)
*High-Ca diet*	1.72 (0.76–3.78)	1.48 (0.72–3.04)

Cox regression models were used

For CKD, model 1 was adjusted for age and sex; model 2 was additionally adjusted for systolic blood pressure, type 2 diabetes, creatinine, body mass index and smoking; model 3 was additionally adjusted for total energy intakes (kcal/d), dietary intakes of total fats (g/d), fiber (g/d)

For HTN, model 1 was adjusted for age and sex; model 2 was additionally adjusted for type 2 diabetes, eGFR, body mass index and smoking; model 3 was additionally adjusted for total energy intakes (kcal/d), dietary intakes of total fats (g/d), fiber (g/d)

Low-, and high-Ca-diet were defined according to median of Ca intakes as 1180 < and > 1180 mg/d, with a median of 990 and 1580 mg/d, respectively

^†^Full model was only reported

Table 4 represents the mean (SD) of eGFR, SBP, and DBP over eight years of follow-up (four examinations) and the estimated overall mean across tertile categories of dietary Ox intakes. Participants with higher intake of dietary Ox (> 220 *vs.* < 150 mg/d) had lower eGFR values over time (75.3, 95% CI = 75.0–76.5 *vs.* 77.3, 95% CI = 76.6–78.1 mL/min/1.73m^2^, *P*_time×group_ = 0.004) and higher SBP levels (112, 95% CI = 111–113 *vs.* 109, 95% CI = 108–110 mmHg, *P*_time×group_ = 0.007).

**Table 4:** Mean eGFR and blood pressures across tertile categories of Ox intake over 8 years of follow-up

	Baseline (2006–2008)	First follow-up (2009–2011)	Second follow-up (2012–2014)	Third follow-up (2015–2017)	Overall mean (95% CI)	*P*_time×group_
eGFR, *mL/min/1.73m*^*2*^						
< 150	81.4 ± 11.9	80.0 ± 12.3	74.6 ± 13.1	72.0 ± 12.1	77.3 (76.6–78.1)	0.004
150–220	80.2 ± 12.1	78.5 ± 12.9	72.8 ± 13.5	70.5 ± 12.9	75.9 (75.1–76.6)	
≥ 220	79.6 ± 12.1	77.9 ± 13.1	73.6 ± 13.2	70.8 ± 12.3	75.3 (75.0–76.5)	
*SBP, mmHg*						
< 150	106 ± 12.1	110 ± 13.3	112 ± 14.2	111 ± 14.3	109 (108–110)	0.007
150–220	108 ± 11.8	111 ± 13.8	112 ± 15.3	112 ± 15.8	111 (100–112)	
≥ 220	108 ± 12.4	112 ± 13.2	113 ± 14.6	113 ± 15.2	112 (111–113)	
*DBP, mmHg*						
< 150	75.5 ± 9.1	75.0 ± 9.8	75.9 ± 10.0	76.0 ± 9.2	74.1 (73.6–74.7)	0.489
150–220	71.5 ± 8.6	75.5 ± 9.8	75.9 ± 9.6	75.5 ± 9.4	74.5 (73.9–75.0)	
≥ 220	71.3 ± 9.1	75.6 ± 10.3	76.3 ± 9.3	76.1 ± 9.1	74.6 (74.1–75.2)	

Data are mean ± SD unless stated otherwise. The generalized estimating equation (GEE) was used

*eGFR* estimated glomerular filtration rate; *SBP* systolic blood pressure; *DBP* diastolic blood pressure

**Fig. 2:**
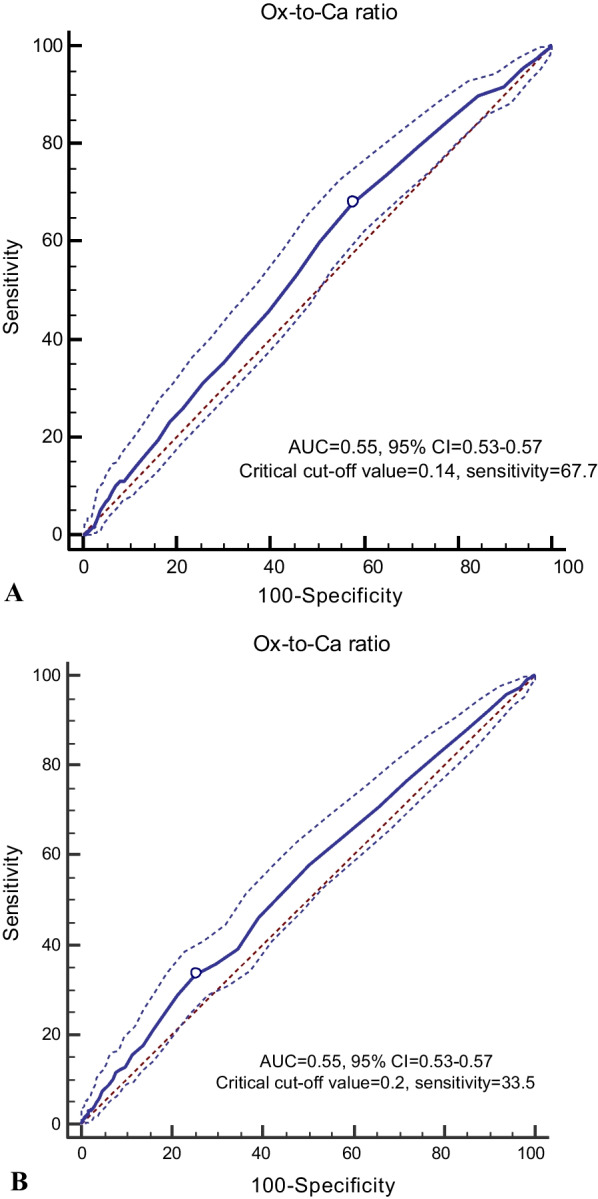
**A** Cut-off point of Ox-to-Ca ratio for incident CKD (0.14, sensitivity = 67.7%, Youden index = 0.10). **B** Cut-off point of Ox-to-Ca ratio for incident HTN (0.20, sensitivity = 33.5%, Youden index = 0.08)

The critical cut-off value of the dietary Ox-to-Ca ratio for HTN and CKD events, as well as sensitivity, specificity, and AUC (*P*-value), are presented in Fig. 2. The critical cut-off point of Ox-to-Ca for predicting CKD was 0.14 (AUC = 0.55, 95% CI = 0.53–0.57, *P* = 0.001; sensitivity = 67.7%, Youden index = 0.10). Cut-off point of Ox-to-Ca ratio for incident HTN was 0.20 (AUC = 0.55, 95% CI = 0.53–0.57, *P* = 0.001; sensitivity = 33.5%, Youden index = 0.08). The Ox-to-Ca ratio cut-off for predicting HTN with a fixed sensitivity value of 80% (i.e., 80% true positive incident case) was 0.11. In the presence of traditional potential risk factors, dietary Ox-to-Ca ratio higher than the cut-off values (≥ 0.14 and 0.20 for CKD and HTN, respectively) were related to an increased risk of developing CKD and HTN by 35% (HR = 1.35, 95% CI = 1.12–1.63), and 22% (HR = 1.22, 95% CI = 1.03–1.45).

## Discussion

In this prospective cohort study of well-characterized men and women with more than eight years of follow-up, we observed that higher dietary Ox intake might increase the risk of developing HTN and CKD, and lower Ca intakes may potentiate the adverse effects of excessive Ox intakes. One potential clinical translation of our findings is that dietary Ox intakes must be balanced with a diet rich in Ca  to prevent the potential adverse effect of dietary Ox. To the best of our knowledge, this was the first population-based prospective study that evaluated the preliminary hypothesis regarding the potential adverse effects of a higher load of Ox and an imbalanced dietary Ox-Ca on the risk of developing HTN and CKD.

The nephrotoxicity of dietary Ox has been long established [11, 14] but has not previously been extended to CKD development, as our results now show. Higher intakes of Ox (328 *vs.* 106 mg/d) were related to a higher relative risk for kidney stones by 22% (HR = 1.22, 95% CI = 1.03 to 1.45) in men; a similar estimated risk (HR = 1.21, 95% CI = 1.01 to 1.44) was also observed in women with higher Ox intake (287 vs. 87 mg/d) [14]. A Ca intake below the median of 755 mg/d potentiated the HR of a high-Ox diet up to 46% (HR = 1.46, 95% CI = 1.11 to 1.93) [14]. Higher intakes of dietary Ox result in elevated Ox excretion [18] and hyperoxaluria [2, 3], situations that are strongly associated with a greater risk of CKD progression; elevated Ox excretion (≥ 27.8 vs. < 11.5 mg/d) increased risk of CKD progression by 33% (HR = 1.33, 95% CI = 1.04–1.70) [12].

No direct evidence is available to connect dietary intakes of Ox to elevated blood pressures. Our findings, however, are consistent with mechanistic data from experimental models indicating toxic effects of Ox on vascular function [43] and with epidemiologic findings reported hypertensive subjects excreted more Ox in urine compared to normotensive subjects (34.8 *vs.* 26.5 mg/day) [44]. Some preliminary evidence implies that Ox accumulation in the human body (exhibited as increased plasma/urine Ox or oxalosis) may adversely affect vascular function [45]. An excessive amount of Ox in the human body induces oxidative stress and inflammation [46, 47], systemic oxalosis, and deposition of Ox in vascular tissues [48, 49], mechanisms that may connect oxalemia and hyperoxaluria with developing both HTN and CKD. Furthermore, a higher load of Ox intakes impacts monocyte cellular bioenergetics and mitochondrial complex activity and activates inflammatory signaling in humans [50]. High-Ox concentrations impair oxidant-antioxidant balance by reducing glutathione levels, increasing reactive oxygen species (ROS) generation, inducing mitochondrial permeability transition mediated cell death, and MCP-1 secretion [5–8], critical events leading to the development of both vascular and renal dysfunction. Increased plasma Ox concentrations lead to accumulated vascular Ox levels, increases serum malondialdehyde (MDA), advanced oxidation protein products (AOPP), and tumor necrosis factor-α (TNF-α) levels, and decreases superoxide dismutase activity [43]. These events are evident as underlying predisposing mechanisms for the development of HTN.

A balanced Ox-Ca diet preventing secondary hyperoxaluria and its complications in healthy humans has not yet been defined. Some preliminary data report that decreased ingested Ca by 60% (from 1002 to 391 mg) in a diet containing 250 mg Ox/day increased the contribution of dietary Ox in urinary Ox excretion by eightfold (from a mean of 6.6 [51, 52] to 53%) [22]. Dietary Ca is recommended to be consumed with Ox-rich foods to maximize the Ox binding effect of Ca in the GI tract and decreased urinary Ox excretion [22].

Some limitations of our study also warrant discussion. Estimation of Ox intakes, as well as other nutrients in our population, were conducted based on the non-national food composition tables, which may be considered a source of error in the estimation of exposure. Furthermore, the lack of data on plasma Ox levels and urinary Ox and Ca concentrations of the study participants was a significant limitation; such data provides more insights into intestinal-renal handling of Ox and the status of Ox homeostasis in the body. Lack of data for plasma uric acid concentrations of the participants and information about kidney stones (as potential confounding variables) that might affect the relationships of dietary Ox with CKD was another limitation. We also could not capture other factors, including rare disorders of Ox metabolism (primary hyperoxaluria), Ox over-absorption (enteric hyperoxaluria), or excessive intake of its precursors (e.g., ethylene glycol poisoning), which may involve in whole-body Ox homeostasis beyond ingestion of Ox. In our study, the definition of T2DM was based on serum glucose and drug information, due to lack of data on glycosylated hemoglobin (HbA1C). Although HbA1C gives an indication of chronic glycaemia rather than being a test of glycaemia at a single point in time [53], population-based studies reported a good agreement between HbA1C and FSG for identifying T2DM [54, 55].

## Conclusion

Our data support the hypothesis that dietary Ox may be a significant nutritional risk factor for incident HTN and CKD. The relation between dietary Ox and risk of HTN and CKD seems to be varied by Ca intake, and subjects with lower Ca intakes may be more burdened by excessive intakes of Ox. Further research is needed to establish the potential effects of dietary Ox concerning other determinants of Ox homeostasis (i.e., endogenous Ox production, urinary Ox excretion) on regulating blood pressure, vascular function, and development of HTN and CKD.

## Data Availability

Data will be presented upon forwarding the request to the corresponding author (z.bahadoran@endocrine.ac.ir) and confirmation of the director of RIES (azizi@endocrine.ac.ir).
